# Childhood obesity prevention in general practice: supporting implementation through co-ideation

**DOI:** 10.1093/fampra/cmad117

**Published:** 2024-01-18

**Authors:** Michelle Gooey, Heather Morris, Michaela Baulderstone, Kristina Flego, Phoebe Holdenson Kimura, Rebecca Kathage, Kylie Rix, Ashraf Saddik, Wei-May Su, Peter Bragge, Heidi Bergmeier, Amanda O’Connor, Elizabeth Sturgiss, Helen Skouteris

**Affiliations:** Health and Social Care Unit, School of Public Health and Preventive Medicine, Monash University, Melbourne, Australia; Health and Social Care Unit, School of Public Health and Preventive Medicine, Monash University, Melbourne, Australia; Oakden Medical Centre, Hillcrest, Australia; Warrandyte Road Clinic, Ringwood, Australia; General Practice Clinical School, Sydney Medical School, University of Sydney, Sydney, Australia; East Canberra General Practice, Majura Park, Australia; Paediatrics Department, Grampians Health, Ballarat, Australia; St Kyrollos Family Clinic, Coburg, Australia; Health Education and Training Institute (HETI HE), NSW Health, St Leonards, Australia; Translational Health Research Institute (THRI), Western Sydney University, Campbelltown, NSW, Australia; School of Medicine, The University of Notre Dame, Australia; BehaviourWorks Australia, Monash Sustainable Development Institute, Monash University, Clayton, Australia; Health and Social Care Unit, School of Public Health and Preventive Medicine, Monash University, Melbourne, Australia; Health and Social Care Unit, School of Public Health and Preventive Medicine, Monash University, Melbourne, Australia; School of Primary and Allied Health Care, Monash University, Frankston, Australia; Health and Social Care Unit, School of Public Health and Preventive Medicine, Monash University, Melbourne, Australia; Warwick Business School, University of Warwick, Coventry, United Kingdom

**Keywords:** Australia, child, general practice, general practitioners, health behaviour, paediatric obesity

## Abstract

**Background:**

Childhood obesity is associated with physical and psychological complications thus the prevention of excess weight gain in childhood is an important health goal. Relevant to the prevention of childhood obesity, Australian general practice-specific, preventive care guidelines recommend General Practitioners (GPs) conduct growth monitoring and promote a number of healthy behaviours. However, challenges to providing preventive care in general practice may impact implementation. In October and November, 2022, a series of three workshops focusing on the prevention of childhood obesity were held with a group of Australian GPs and academics. The objective of the workshops was to determine practical ways that GPs can be supported to address barriers to the incorporation of obesity-related prevention activities into their clinical practice, for children with a healthy weight.

**Methods:**

This paper describes workshop proceedings, specifically the outcomes of co-ideation activities that included idea generation, expansion of the ideas to possible interventions, and the preliminary assessment of these concepts. The ecological levels of the individual, interpersonal, and organisation were considered.

**Results:**

Possible opportunities to support childhood obesity prevention were identified at multiple ecological levels within the clinic. The preliminary list of proposed interventions to facilitate action included GP education and training, clinical audit facilitation, readily accessible clinical guidelines with linked resources, a repository of resources, and provision of adequate growth monitoring tools in general practice.

**Conclusions:**

Co-ideation with GPs resulted in a number of proposed interventions, informed by day-to-day practicalities, to support both guideline implementation and childhood obesity prevention in general practice.

**Key messages**

- Multiple opportunities to support obesity prevention exist in general practice.
- Potential research interventions can be identified through co-ideation.
- Collaborative research with general practitioners is feasible and achievable.

## Introduction

In Australia, approximately one in four children (2–17 years old) are living with either overweight or obesity.^1^ Globally, it is predicted that the number of children aged 5–19 years old who are living with obesity will rise from 158 million in 2020 to 254 million in 2030.^2^ Childhood obesity is associated with physical and psychological complications such as hypertension, impaired glucose tolerance, depression and anxiety, and as well as adult diseases such as coronary artery disease and some cancers.^3^ Furthermore, children already living with obesity are more likely to have ongoing obesity into adulthood. Hence preventing excess gain and maintaining a healthy weight in childhood can have important implications for both current and future health.

Preventive care is a vital activity in Australian general practice and General Practitioners (GPs) play a central role, not only in the treatment of obesity but also in prevention. The Royal Australian College of General Practitioners (RACGP) “Guidelines for preventive activities in general practice”^4^—more commonly known as the “Red Book”—is considered to be “the” guide for preventive care for Australian general practices. Consistent with recommendations in international clinical practice guidelines for the prevention of obesity amongst children,^5^ the Red Book recommends GPs conduct growth monitoring and promote a number of healthy behaviours throughout childhood (see Supplementary Table).^4^

We are not aware of recent data assessing the frequency of obesity-related preventive care activities amongst Australian GPs, however, GPs in Australia agree they can play a role in providing general preventive care to children.^6,7^ Challenges to providing such care include time pressures^6,7^; inadequate GP remuneration^6,7^; out-of-pocket costs for families^6^; a need for suitable resources^7^; and considerations of appropriateness when a child is acutely unwell.^7^ These challenges are consistent with a broader recognition that barriers commonly disrupt clinical guideline implementation.^8^ Specifically, in the area of childhood obesity prevention, a need for implementation tools to accompany existing clinical practice guidelines has been highlighted.^5^

The context or setting for implementation and the barriers and facilitators within it are critical considerations in the translation of research into routine practice.^9^ To build understanding of childhood obesity prevention in the real-world context of Australian general practice, a series of three collaborative workshops were convened in late 2022. The objective of the workshops was to determine practical ways that GPs can be supported to address barriers to the incorporation of obesity prevention activities into their clinical practice, with a particular focus on children with a healthy weight.

## Methods

### Background

The workshops were undertaken as part of a larger project underpinned by the Intervention Mapping process,^10^ which is recognised as a beneficial methodology for developing behaviour change interventions amongst healthcare professionals.^11^ The Intervention Mapping process emphasises the importance of partnerships and participatory action with stakeholders,^10^ thus the workshops were designed to foster collaboration between practicing GPs and academics for intervention development.

### Participants

Seven GPs (MB, KF, PHK, RK, KR, AS, MS) joined the workshops in response to an email invitation distributed via professional networks. These GPs were from four different Australian states and territories—six from a capital city and one based in a regional area—and two GPs had a specific paediatric clinical focus. Six GPs attended all three workshops and one GP attended two workshops and then a separate, catch-up session. To recognise the contribution and commitment required of these GPs, an honorarium of $100 an hour (in the form of a voucher) for workshop participation was offered. Seven academics (MG, HS, HB, PB, ES, HM, and AO) with expertise in implementation science, Intervention Mapping, behaviour change, general practice, and/or public health joined the workshops as facilitators (MG, HM, and AO), contributors (PB, ES), and/or observers (HS, HB).

### Workshop description

The workshops were held on 10th October, 7th November, and 28th November 2022. To facilitate attendance by GP members, the workshops were held in the evening and conducted virtually via video meeting. Each workshop was strictly limited to 2 h in duration, given that all attendees were busy professionals giving up their personal time to join the workshops outside of standard business hours. The workshops were facilitated by MG with HM or AO.

The workshops consisted of two phases. The “setting the scene” phase will not be described in detail as it is beyond the scope of the paper, however, it sought to establish a common understanding of how GPs and general practices might carry out obesity prevention in clinical practice and identify the barriers to doing this and how these might be overcome. The focus of this paper is the “co-ideation” phase. Co-ideation is described by Pearce et al. as “engaging in open dialogue to share new and creative ideas for the solving of problems relating to new products, services, policies and programs”.^12^ The co-ideation phase consisted of three activities that explored possible interventions at the ecological levels of the individual (the GP), interpersonal (patients, their family, and colleagues), and organisation (the clinic) to encourage more growth monitoring and healthy behaviour promotion consistent with the RACGP Red Book. The three co-ideation activities were:

1. Idea generation: Divided into two groups, GPs brainstormed how to support more routine growth monitoring and promotion of healthy behaviours, with particular consideration of different ecological levels.
2. Idea expansion: the group reviewed the ideas generated in the previous activity, grouped by commonalities. Building on these ideas, the GPs determined a list of proposed interventions that could be implemented.
3. Assessing the interventions: using a shared online, customised spreadsheet, the GPs individually assessed each of the proposed interventions considering utility, impact, feasibility, sustainability, and relative advantage using a Likert scale of 1 to 5 (score of 1 = not at all and 5 = very much), an adapted version of the “NUF test”.^13^

### Data

In this paper, we report on the discussions and outcomes of the co-ideation process for supporting childhood obesity prevention in general practice. Proceedings of the workshops were captured during the workshops through online documents and facilitator notetaking in real time. The workshops were recorded to verify and augment meeting notes after each workshop and all attendees of the workshops are co-authors of this report. Outcomes from the “Idea generation” activity were collated and summarised according to commonalities by MG, as presented below, between workshops 2 and 3; these were the starting point for discussion in the “Idea expansion” activity. Products of the “Idea expansion” activity are listed with a summary of key discussion points. In the final activity, “Assessing the intervention”, the total score for each GP’s quantitative assessment of each idea was summed to create a final group score for each proposed intervention to enable relative ranking.

## Results

The following sections describe the output of each of the co-ideation activities.

### Idea generation

Commonalities relating to the ideas generated in this activity are summarised in Table 1, according to ecological level. Although ideas for growth monitoring and healthy behaviour promotion were brainstormed separately, some consistency was seen at each ecological level across both topics.

**Table 1.** Commonalities for supporting growth monitoring and promotion of healthy behaviours, according to ecological level, in the Idea generation activity.

		Growth monitoring	Promoting healthy behaviours	Growth monitoring and promoting healthy behaviours
Ecological level	Individual: GP	Improve consistency of related information	Encourage sensitive discussions	Increase knowledge and skills Improve access/knowledge of where to find appropriate tools and resources Provide strategies to support implementation
	Interpersonal: patient/family/clinic colleagues	Encourage families to participate in growth monitoring Involve colleagues in growth monitoring	Promote general practice as a source of preventive care Work with families to find out what they want	Provide relevant information and resources to families
	Organisational: Clinic			Create a supportive environment Facilitate the availability of helpful tools Take a whole clinic approach

At the level of the individual GP, commonalities of brainstormed ideas across both growth monitoring and promoting healthy behaviours were: the need to increase knowledge and skills; ensuring access to the right tools and resources and knowledge of where to find them; and providing strategies that might best support implementation into routine practice. Discussed examples of further education included monitoring growth trajectories, taking a holistic approach to growth, improving skills such as motivational interviewing, and finding appropriate conversational starters. The group also noted that clinicians’ understanding of growth monitoring can be diverse, and interventions to promote a more consistent approach amongst clinicians were identified as lacking. It was also highlighted that whilst relevant clinical resources are available to support growth monitoring and promotion of healthy behaviours, it might be difficult for some GPs to locate suitable resources from a trusted education source. Therefore, improving knowledge of key resources may be helpful. With respect to promoting healthy behaviours, the need for awareness about the importance of having sensitive discussions and how this could be done, such as through non-stigmatising language and non-judgmental attitudes, was also emphasised. Concerns regarding possible harms, for example relating to the broader complexities of obesity and disordered eating, were acknowledged.

At the interpersonal level, the need for specific materials targeted at families was identified, for example, booklets that frame children engaging in healthy behaviour in a positive way or posters in the waiting room promoting growth monitoring. The group also highlighted the importance of increasing awareness of preventive care provision in general practice to improve acceptance and engagement of healthy behaviour promotion amongst families. With regard to growth monitoring, in particular, the involvement of other clinic colleagues such as nurses or reception staff to support this activity was also suggested.

At the level of the clinic operations—the organisational level—the group discussed the need to create a supportive environment within the clinic to carry out these activities, facilitate availability of the right tools, and take a whole clinic approach for both growth monitoring and promoting healthy behaviours. Specific ideas included “check-up” style appointments for school-aged children, discussion of audits of routine growth monitoring at clinic meetings, and whole-clinic training with respect to using sensitive language and avoiding weight stigma.

### Idea expansion

The preliminary list of interventions proposed by the GPs (Table 2) was as follows:

**Table 2.** Summary list of proposed interventions to support childhood obesity prevention in general practice in the Idea expansion activity.

Education and training Facilitate clinical audits Readily accessible guidelines with linked resources Repository of resources Provision of growth monitoring tools

- *Education and training:* Online and/or face-to-face modules for GPs, covering both growth monitoring and promoting healthy behaviours. The building of skills to conduct these activities in a mindful and sensitive way to promote positive interactions between the GP, child, and their family was considered critical.
- *Facilitate clinical audits:* Materials to support GPs and practices wanting to conduct clinical audits of growth monitoring activity. This is particularly topical due to recent changes to continuing professional development for medical practitioners in Australia, which requires not only the inclusion of educational activities but also those that relate to reviewing performance and measuring outcomes.^14^
- *Readily accessible guidelines with linked resources:* Clinical guidelines containing embedded links to useful information and hosted on platforms often accessed by GPs and that already hold similar guidelines, for example, the local HealthPathways website. HealthPathways is an online service, managed by the relevant Primary Health Network, which contains guidance for a number of conditions that can be tailored to the local context and is targeted towards primary care clinicians.^15^
- *Repository of resources:* Collection of existing resources into a single repository to improve GPs’ awareness and access to what is already available.
- *Provision of growth monitoring tools:* Facilitate ready-access to tools required to measure paediatric patients’ height and weight for every GP in a given practice to encourage routine use

### Assessing the interventions

Following the assessment, the provision of appropriate growth monitoring tools was the highest-ranking idea. The ranking of the remaining four proposals were clustered with relatively similar final scores.

## Discussion

This paper reports the co-ideation outcomes of three workshops focusing on how Australian GPs can be supported to incorporate childhood obesity prevention activities into their clinical practice. A preliminary list of five interventions was determined by the group: a GP education program, support for clinical audits, clinical guidelines with embedded links to relevant information and hosted on web platforms commonly accessed by GPs, a repository of existing resources, and provision of tools needed for growth monitoring.

The involvement of primary healthcare professionals in childhood obesity prevention is not a new concept. For example, a meta-analysis of four healthcare-based obesity prevention programmes for infants and their parents delivered by midwives or in nurse-led child health clinics in Australia and New Zealand demonstrated initial positive outcomes.^16^ However, effects waned within 1.5–2 years of the interventions ending suggesting a need for interventions to be delivered and/or reinforced throughout childhood.^17^ Furthermore, a review of childhood obesity prevention interventions in primary care indicated that historically the focus has more often been on infants and pre-schoolers rather than an older cohort of children, a relative lack of studies relating to obesity prevention compared to treatment and the urgent need for further research.^18^

In terms of encouraging behaviour change amongst health professionals, reviews have found that, in general, interventions that include audit and feedback, educational programmes, or clinical support decision tools may be of particular value.^19,20^ Furthermore, multi-component interventions are most likely to be successful,^19–21^ suggesting that implementation of just one of the proposed interventions will not be a “magic bullet”. Rather, a comprehensive programme incorporating a combination of proposed ideas is likely to have a greater chance of success.

The final list of interventions from these workshops focused primarily on the ecological level of the individual GP—perhaps reflecting that GPs may feel more comfortable suggesting interventions that they can carry out as individuals rather than assuming that the practice might be able to do more. However, given the ideas and discussions raised in the “Idea generation” activity, there is clear scope to further explore interventions for the interpersonal and organisational ecological levels. Such direction is supported by evidence indicating changes at an organisational level are effective for preventive care implementation^21,22^ and that at the interpersonal level, practice nurses^23^ and other practice staff could also play an important role.^21^

Nevertheless, it is also acknowledged that actions within general practice alone cannot address obesity in Australia. Although these workshops focused on the ecological levels within a clinic, upstream levels such as community and public policy are also critical components of the ecological model that can influence behaviour.^24^ For example, despite the Red book recommendations to provide preventive care throughout childhood,^4^ we are not aware of any formalised government policies with the aim of providing such care to all children in Australian general practice. A government-funded Health Assessment is currently available for Aboriginal and Torres Strait Islander people of all ages,^25^ however, the more widely available government-funded “Healthy Kids check” was discontinued in 2015.^26^ Furthermore, this check was only available for pre-school-aged children and although calculation of body mass index and plotting on a centile chart was a mandatory requirement of the check, other assessments relevant to obesity prevention recommended in the Red Book, such as reviewing eating and physical activity habits, were not.^27^

Additionally, any efforts in the general practice setting should be part of a whole of society, systems-based approach including cross-sectoral action and supportive environmental changes.^28^ Examples of public policy approaches to address obesity include physical activity and healthy eating promotion in schools and taxation of sugar-sweetened beverages.^29^

### Implications for research and practice

These workshops were part of a larger research project exploring childhood obesity prevention in clinical practice. The output will directly inform future work focused on supporting childhood obesity prevention in Australian general practice and the feasibility of taking these ideas forward will be explored.

More broadly, we have shown that GPs and researchers can collaboratively work to address real-world issues, informed by the day-to-day practicalities in general practice. Consistent with the concept of generating new ideas through co-ideation,^12^ there were no pre-determined intervention ideas prior to beginning the workshop series, and the final proposed list of interventions was directly derived from workshop discussions. Whilst the opportunity to provide insights from the field in the initial ideation phase is recognised by this group as uncommon, we believe it is an important step towards a more inclusive service or resource design process and generating new knowledge.

Customising interventions to address identified barriers, as done in these workshops, is also important, particularly in the context of guideline implementation.^19^ Such collaborative approaches could also be applied beyond childhood obesity prevention to address other gaps between guidelines and clinical practice, in a tailored and clinician-centred way.

Finally, as noted previously, healthy weight maintenance has important health implications given the potential long-term complications of obesity in childhood. Furthermore, the clinical benefits of improving the implementation of healthy behaviour promotion and growth monitoring can extend beyond obesity prevention—for example, potentially influencing mental wellbeing^30,31^ and informing diagnosis of other health conditions,^32^ respectively.

### Strengths and limitations

A key strength of these workshops was the collaborative approach between academic and GP members of the group. Reflecting on the workshops, the GPs found the process to be collaborative and professionally enriching. Other strengths of this project included the range of our collective knowledge, consistently high attendance at all three workshops, and the direct application of practical insights, which was underpinned by Intervention Mapping methodology.

The main limitation was the amount of time available in the workshops. However, the duration of the workshops was determined based on pragmatic considerations from the outset. We also recognise that the seven GPs who joined these workshops are unlikely to be a representative sample of Australian GPs, however, the depth of expertise, practical knowledge, and engagement in the topic was of particular benefit to the co-ideation process. Finally, we acknowledge that the NUF tool, which we modified for our own use, is not a standardised academic methodology,^33^ however, it enabled us to make a relative comparison of the proposed interventions quickly and considering a number of factors.

## Conclusion

The workshops delivered important insights regarding how childhood obesity prevention can be supported in general practice, considering the practicalities and existing challenges faced by GPs, and yielded promising ideas for interventions to promote guideline implementation. The preliminary list of proposed interventions included GP education and training, clinical audit facilitation, readily accessible guidelines with linked resources, a repository of resources, and the provision of adequate growth monitoring tools. Planned next steps will consider how the workshop proposals may be prioritised and tested in the future.

## Supplementary Material

cmad117_suppl_Supplementary_Material

## Data Availability

Data are not available to access.
