# Supplementary material for: Childhood obesity prevention in general practice: supporting implementation through co-ideation
Source: Fam Pract. 2024 Jan 18;41(1):25–30. doi: 10.1093/fampra/cmad117 (PMC10901475; doi:10.1093/fampra/cmad117)
Supplement: cmad117_suppl_Supplementary_Material [file cmad117_suppl_supplementary_material.pdf]

Supplementary table:

Summary of growth monitoring and promotion of healthy behaviours relevant to obesity prevention in childhood from the "Guidelines for preventive activities in general practice"[1]

Age	Growth monitoring	Promotion of healthy behaviours
Neonatal	Physical examination per the Child Personal Health Record (or equivalent)	
2, 4, 6, 12 and 18 months; and 3 years	Physical exam per the Child Personal Health Record:<br>• regular measurement, plotting on growth charts and interpretation<br>• include body mass index (BMI) from 2 years	Support breastfeeding<br>Promote healthy eating (second year of life), physical activity and sleep
3.5–5 years	Physical exam (include BMI)	Promote healthy eating, drinking, physical activity and healthy sleep
6–13 years	Routinely measure growth and BMI	Promote healthy eating, drinking, physical exercise and sedentary behaviour reduction
14–19 years	Routinely measure growth and BMI	Promote healthy eating, drinking, sleep and physical activity

Extracted from Table 3.1: Age-related health checks in children and young people in the "Guidelines for preventive activities in general practice"[1]

Reference:

1. The Royal Australian College of General Practitioners. Guidelines for preventive activities in general practice. 9th ed, updated. East Melbourne, Vic, Australia: RACGP; 2018
